# Resilience, sense of danger, and reporting in wartime: a cross-sectional study of healthcare personnel in a general hospital

**DOI:** 10.1186/s12960-023-00866-w

**Published:** 2023-10-11

**Authors:** Sarah Sberro-Cohen, Inbal Amit, Erez Barenboim, Alona Roitman

**Affiliations:** 1 Maccabi Health Care Services, Omer, Israel; 2 Assuta Ashdod Hospital, Ashdod, Israel

**Keywords:** Resilience, Sense of danger, Wartime, Attendance at work

## Abstract

**Background and Aims:**

Maintaining healthcare services and ensuring the presence of healthcare personnel (HCP) during periods of conflict and high-intensity warfare in Israel including the significant security event that occurred on May 2021, pose significant challenges for hospitals in the range of missile attacks. The May 2021 event, marked by intense hostilities and military actions, brought about heightened security escalations and increased risks in the region. Despite the prevailing threat of missile attacks and ongoing security concerns, hospitals in the affected areas were required to sustain their services and uphold care standards. In light of these circumstances, this study aims to identify the factors that influence the percentage of HCP reporting for work during these intense periods of security escalations and wartime in Israel. Specifically, it explores the relationships between resilience, sense of danger, and HCP absenteeism in the context of the ongoing conflict. The findings of this study can provide valuable insights for designing interventions aimed at decreasing HCP absenteeism during security escalations, wartime, and emergency situations, ultimately contributing to the resilience and effectiveness of healthcare delivery in this challenging environment.

**Methods:**

During a relative calm period from December 2021 to January 2022, a cross-sectional study was conducted at a southern Israeli general hospital, situated within the range of missile attacks in the midst of a longstanding conflict. The study focused on HCP who were employed before May 21, which marked the end of the last war state at that time. The questionnaire, consisting of measures for resilience using the Conor-Davidson scale (CD-RISC 10) and the sense of danger assessed with the Solomon & Prager inventory, was administered online to all hospital employees at Assuta Ashdod Hospital, located in the southern city of Ashdod, Israel. This approach was chosen due to the challenging nature of conducting a study during an existing war, making it impractical to carry out the research during such periods of active war.

**Results:**

In total, 390 employees completed the survey (response rate of 24%). Of this sample, 77.4% reported fully to work during the last security escalations in May 2021. Most of the sample (84.1%) felt insecure on the way to work. The HCP who reported fully to work had a higher level of resilience than employees who reported partially or did not come to work at all (*p* = .03). A higher sense of danger in the workplace correlated with a 73% increase in absenteeism (*p* < .01). Absenteeism (partial or full) was higher among HCP with children who require supervision (*p* < .01). Hospital preparedness for emergencies as perceived by the employees increased HCP attendance at work (*p* = .03).

**Conclusions:**

Hospital management should consider designing programs aimed at potentially strengthening the level of resilience and fostering a greater sense of security among hospital personnel, which might encourage greater attendance at work during wartime, crises, or emergencies.

## Introduction

Since its inception, the State of Israel has faced security challenges, with sudden, abrupt transitions from daily routine to states of emergency and back. During periods of escalation and wartime, hospitals situated in areas within the zone of fire must cope with numerous challenges. They assume a critical role in providing immediate medical care and support to those injured or affected by the ongoing conflicts, while also contending with significant obstacles such as the risk of missile attacks, bombings, and security threats. In order to ensure optimal functioning in emergencies, and optimal performance on the part of healthcare personnel, managers need to demonstrate leadership and personal involvement while providing clear guidelines [1]. Along with the challenge of treating patients with physical and mental injuries, the greatest hurdle involves maintaining staff safety and making sure that they report for work to fully address the needs of patients during the emergency. Hospitals at the forefront of the fighting are required to be ready to handle casualties beyond their routine duties and swiftly respond to emergencies Nevertheless, hospital employees may fail to show up for work in an emergency for a variety of reasons [2]. The willingness of teams to report for work in times of disaster and emergency depends on the type of incident [3]. Studies in Israel have found that Israeli nurses were less willing to report for work during periods of terrorist threats. The higher the sense of threat, the lower the nurses’ willingness to report for work [4]. Attendance was reported to be the highest in the case of natural disasters (61.9%), followed by seasonal flu (52.5%), smallpox (47.8%), and the SARS /COVID-19 pandemic (43.5%), compared to mass shooting incidents (37.5%), chemical warfare/bomb threats (31.9%), and biological warfare (28.17%). The most common reasons for absenteeism were found to be distrust, and a lack of sense of security for health care workers and their families [5]. In emergency events such as pandemics healthcare worker absenteeism can be attributed to family responsibilities, such as childcare and elderly care, along with increased stress and burnout from working long and exhausting hours [6]. Maslow’s pioneering work (1943) argued that humans have a strong drive to guarantee their own safety. Safety in terms of one’s physical existence is defined as the second core level in the pyramid of needs, immediately after basic physiological needs [7]. Alderfer’s ERG theory and Herzberg’s two factor theory of motivation also posit that lower order needs must be met first to increase motivation and prevent job dissatisfaction. Alderfer's ERG theory suggests that there are three groups of core needs: existence (E), relatedness (R), and growth (G); hence the acronym ERG. These groups align with Maslow's levels of physiological needs, social needs, and self-actualization needs, respectively [8]. Thus, when there is an escalation in threat level, the need for security is of utmost importance for healthcare personnel, and extends to their family members. Medical institutions must respond to this need for security to ensure staff commitment in times of emergency [9]. Studies have shown that a high sense of danger and concerns about potential threats are associated with low resilience, a lesser ability to recover, and high stress [10].

Resilience is defined as the ability to bounce back from adversity, and to draw strength from this recovery [11]. Studies have shown that there is a significant positive relationship between community, national and individual levels of resilience [12]. Community resilience refers to an entire community's ability to recover from a difficult event and return to everyday life as quickly as possible. The definition of national resilience is broader and refers to a society's sustainability and durability with regards to issues in diverse fields [13]. Individual resilience is defined as the individual's ability to successfully cope with difficult events (such as disasters, wars, etc.) and return to one’s level of previous functioning in as short a time as possible [14–16]. The American Psychological Association (APA) defined individual resilience as a person's ability to adapt positively to new life circumstances following exposure to a traumatic event, tragedy, threats, or other significant sources of stress [17]. The ability to function effectively in stressful situations at work is significantly influenced by employees' individual resilience [18]. However, employees who experience mental exhaustion need emotional support to be able to face the hurdles that can lead to absenteeism [9]. The COVID-19 pandemic showed the importance of appropriate emotional support to staff by health care organizations [19], where leadership that encouraged emotional and cognitive structuring helped strengthen individual resilience [20].

The current study was designed to identify the predictive factors in HCP to report for work during wartime. Specifically, it examined the relationship between individual resilience and sense of danger and HCP absenteeism in wartime. It was hypothesized that attendance during wartime would be higher in HCP with higher levels of resilience and lower levels of sense of danger.

## Method

### Design

This cross-sectional study was conducted from December 2021 to January 2022 at Assuta Ashdod Hospital, a public hospital located in Ashdod, a peripheral city in southern Israel, 40 km from the Gaza Strip. The decision to conduct the study during a relative calm period was in response to the challenges of conducting real-time research during times of war. This city has been under intermittent missile attacks for nearly a decade and a half. Assuta Hospital is fortified except for two wards. During periods of security escalations and wartime, the patients are moved from the unfortified areas to the fortified ones.

### Participants

The sample was composed of healthcare personnel (physicians, nurses, nursing assistants and medical transportation personnel, health professionals, maintenance and administration employees and others), who met the following criteria: on the hospital staff since at least May 2021 (the previous major security escalations). The exclusion criteria were outsourced employees and students. Recruitment took the form of convenience sampling. All HCP (approximately 1600 employees) were contacted by email through the hospital system and asked to voluntarily complete an online survey.

### Measures

The online questionnaires examined individual resilience, sense of danger, and presence/absence at work during wartime. All the participants filled in general demographic information including their professional status and other background variables. All questionnaires were anonymous.

**Individual Resilience** was measured on the abridged Hebrew version of the Conor-Davidson scale (CD-RISC) [21], validated by Campbell & Stein. The CD-RISC-10 consists of 10 statements describing different aspects of resilience (flexibility, self-efficacy, ability to regulate emotion, optimism and maintaining attention under stress), which are rated on a 5- point Likert scale from 0 (*not at all true*) to 4 (*true nearly all the time*). The total score thus ranges from 0 to 40. Higher scores suggest greater resilience and lower scores suggest less resilience, or more difficulty bouncing back from adversity [22]. The Cronbach alpha for reliability was α = 0.902.

**Sense of danger** was evaluated on the six-item Solomon & Prager Sense of Danger scale [23] that measures personal, family, workplace, and homeland aspects of danger (questions 1–4) (e.g., "During wartime events I feel that my life is in danger"). Terrorism was evaluated using two questions taken from the Eshel et al. scale (questions 5–6) [24] (e.g., "During wartime my daily life is upended "). The items were rated on a Likert scale from 0 (*not true at all*) to 4 (*almost always true*). The scale’s reliability in the present study was α = 0.849.

**Reporting for work during wartime** was evaluated on three items which measured the frequency of reporting for work during the last security escalations (fully/partially/not at all), factors that make it difficult to report for work, and one open question about factors that affect reporting for work.

**Concerning issues** relevant to wartime, the study employed binary response questions (i.e., "yes" or "no") to assess factors such as the impact from previous terrorist incidents, workplace support (including participation in support workshops for employees), and the utilization of the day care centers operated by the hospital.

### Data analysis

Analyses were performed using the SPSS statistical package (IBM Corp. 2019. IBM SPSS Statistics for Windows, version 28.0. Armonk, NY: IBM Corp). Continuous variables are presented as the mean ± standard deviation (SD), whereas the dichotomous and categorical variables are presented as percentages and frequencies. Comparisons used an independent t-test, a Mann–Whitney U test or a Chi square test, as appropriate. Then, a multivariate logistic regression model using the backward stepwise selection method was applied to assess the relationship between full attendance at work during escalation and war and the set of potential predictive factors. All statistical tests were two-tailed and statistical significance was set at* p* < .05.

## Results

### Participants

The final sample was composed of 390 employees of Assuta Ashdod Hospital for a response rate of 24%. Data were collected over a period of 23 days from December 2021 to January 2022. Participants ranged in age from 21 to 73. Among the participants, 77% were females, representing 23% of all women in the hospital, while 23% were males, accounting for 17% of all men in the hospital. Table 1 presents the demographic characteristics. Most participants were married or lived with a partner, and tended to be secular. Almost half had children between the ages of 2 and 10.

**Table 1:** Demographics

Variable		n	%
Age	21–40	212	56.4
	41–60	156	41.5
	61+	8	2.1
Gender	Female	300	76.9
	Male	90	23.1
Family status	Single	36	9.2
	Married/Partner	317	81.3
	Divorced/Widow	37	9.5
Children’s age	Below age 2	60	15.4
	2–10	176	45.1
	10–17	126	32.3
	18+	57	14.6
Religion	Jewish	350	89.7
	Muslim/Christian/Other	39	10.3
Religiosity	Secular	253	64.9
	Traditional	76	19.5
	Orthodox	45	11.5

The sample mainly consisted of nurses (44.9%), health professionals (15.4%) and physicians (13.6%). Of these, 83.1% had a BA/B.Sc. or higher. Almost half of the participants had 1–9 years’ seniority (Table 2).

**Table 2:** Job profiles

Variable		n	%
Education	High school	66	16.9
	BA/ B.Sc	324	83.1
Management position	yes	96	24.6
	no	294	75.3
Profession	Physician	53	13.6
	Nurse	175	44.9
	Nursing assistant and medical transportation personnel	43	11
	Health professionals	60	15.4
	Maintenance and Administration	39	10
	Other	20	5.1
Seniority	1 year or less	7	1.9
	1–9	188	51.6
	10–19	111	30.4
	20 years or more	58	15.9

### Attendance at work in wartime

Most of the sample (77.4%, n = 302) reported for work during the last security escalations in May 2021. The average age of the HCPs who reported for work was higher (41.7 years) than the average age of the HCPs who had some absenteeism or did not report at all (36 years) (p < .01). The study found a significant relationship between children's ages and the degree of reporting to work. Specifically, 84.4% of the HCP who fully reported to work during wartime had no children under the age of two (*p* < .01). Specifically, fewer than half (42.57%) of all parents of children aged 2 to 10 took advantage of the employees' childcare framework operated by the hospital during the time when educational frameworks were closed due to the emergency situation. Only 6.4% (n = 25) of the sample participated in emotional support sessions with a hospital psychologist or social worker, whereas slightly more 14.4% (n = 56) participated in group activities at work for emotional ventilation. Hospital preparedness as perceived by the employees for emergencies increased HCP attendance (*p* = .03). Among the physicians who reported fully for work during wartime (Table 3), this group included specialists (93.8%) and those in internal medicine (71.4%). The rate for full attendance among nurses was 81.7%, whereas laboratory workers reported an attendance rate of 80%, medical transportation personnel reported a rate of 71.4%, medical technicians reported for work at a rate of 66.6%, social services personnel reported at a rate of 60%, and only 40% of the pharmaceutical staff reported for work.

**Table 3:** Attendance by profession

Profession	N	No absenteeism % (N)	Absenteeism (partial or full) % (N)
Physicians	53	84.9% (45)	15.1% (8)
Nurses	175	81.7% (143)	18.3% (32)
Nursing assistants and medical transportation personnel	43	81.4% (35)	18.6% (8)
Health professionals	60	65% (39)	35% (21)
Maintenance & Administration	39	69.2% (27)	30.8% (12)
Other	20	65% (13)	35% (7)

#### Resilience

The mean score for resilience on the CD-RISC 10 was 28.9 (SD 5.8), resilience in HCPs who reported for work was higher than in HCP indicating partial or total absenteeism (*p* = .03).

#### Sense of danger

Sense of danger was lower among HCPs with no absenteeism than HCPs indicating partial or full absenteeism (p = .004). Of the sample, 86.9% (n = 339) indicated feeling insecure on the way to work (81.5% came to work by car).

### Multivariate analysis

**Fig. 1:**
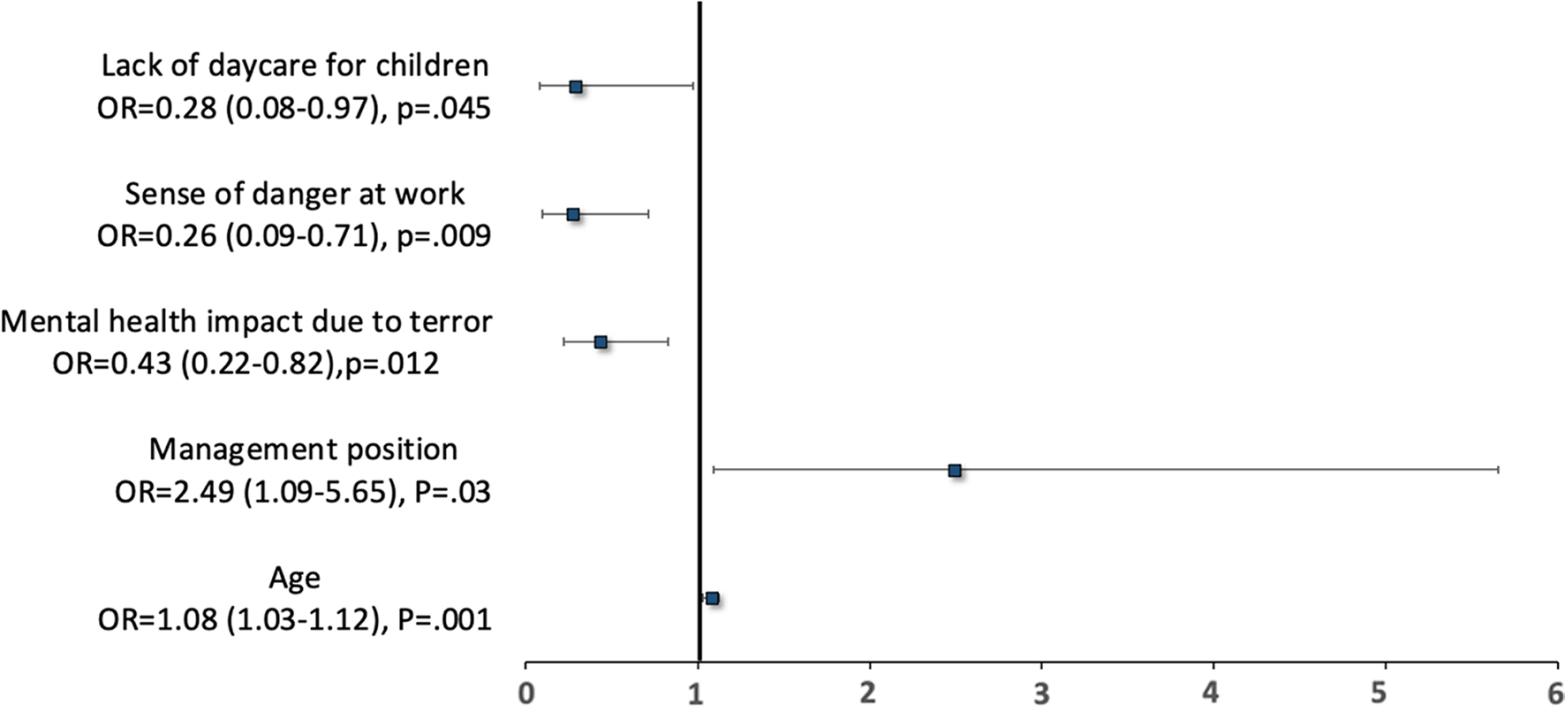
Multivariate model

To better understand the relationship between the dependent variable of 'reporting for work during wartime’ and the independent variables, we ran a multivariate model analysis. The results (Fig. 1) indicated that having a management position increased the likelihood of reporting for work by 2.4 fold (*p* = .03), and that being older also increased reporting for work (p = .001). By contrast, three factors increased absenteeism. A higher sense of danger in the workplace reduced the tendency to report by 73% (*p* < .01) as did a lack of regular daycare for children (*p* = .045), and the impact of terror on mental health (*p* = .012).

## Discussion and conclusion

This study explored the relationships between resilience, sense of danger and attendance at work in wartime, aiming to identify predictive factors in the HCP of a general hospital located in a city that was under multiple missile attacks in southern Israel.

The findings suggest that the level of resilience was associated with the rate of absenteeism in HCP during wartime. One of the major components of resilience is an adaptive capacity, which enables the individual to cope with challenges, changes and risk and can promote better quality of care [25]. The sample’s resilience mean score was lower (28.9, SD = 6.8) than figures reported for the general population in the US (32.1, SD 5.8) [19], Brazil (29.1, SD 5.5) [26], and Spain (29.0, SD 0.1) [27]. HCP resilience was also low as compared to reports for surgeons in the US (33.4, SD 4.0) [28] or US nurses in the aftermath of a tornado (36.7) [29]. Resilience is important for organizations, especially those that deal with saving lives such as hospitals and health care services. It is not only important to have high resilience in times of war, but also when dealing with pandemics [30]. Resilience in emergency management is now considered part of the core agenda in both research and practice. Establishing a proactive vision of resilience and recognizing the complex nature of current systems could enhance managers’ ability to coordinate resilient performance in healthcare.

Currently, businesses and organizations often incorporate courses that focus on personal and organizational resilience as part of their training programs in employee management. The response to the COVID-19 pandemic provides a good example of the range of measures implemented by organizations to help their employees deal with the hardships that characterized that period. These included taking the difficulties of parents of small children into account, options for working from home, divisions into shifts with occupational flexibility, and holding team meetings.

One noteworthy finding from our study is the relatively low utilization of the hospital's childcare framework during wartime. The lack of daycare services for younger children might have contributed to the reduced attendance of this specific group of employees during wartime.

This finding highlights the importance of providing accessible and supportive daycare services for employee children, as it can significantly impact their attendance and, consequently, overall workforce continuity. Offering adequate daycare facilities may encourage more employees to utilize this service, allowing them to attend work and contribute to the hospital's ability to provide health services during wartimes.

Additionally, our study revealed that the majority of our sample feels a sense of danger on their way to work. This underscores the need for decision-makers to develop and implement plans to improve the sense of security during the healthcare professionals' commute. Ensuring safer transportation options and addressing any potential safety concerns on the routes to work can significantly enhance the attendance of HCPs, who are essential workers during wartime. Their collective efforts, including healthcare personnel in transportation, maintenance, administration, and various other roles, are indispensable in providing continuous and comprehensive healthcare services, ensuring hospital operations, and supporting the well-being of patients in times of war.

Overall, the current findings can be harnessed by management to design interventions for employees to better mobilize them to cope with future crises with less absenteeism. Future research should examine other hospitals and community health services to better understand the comprehensive structure of resiliency and formulate recommendations for potential interventions to promote safety.

### Limitations

There are several limitations to this study. This study was only conducted in one hospital, which prevents generalization to other hospitals in Israel and other countries. There is a possibility of selection bias, as the sample may not fully represent all HCPs in the hospital due to voluntary participation. During a period of "relative calm," the study assessed resilience and sense of danger retrospectively, 7 months after the previous security escalations. As a result, it is likely that measuring these factors during or immediately after a significant wartime event would have revealed different data and conclusions. However, the current findings can be utilized by organizations to establish a baseline for HCP absenteeism during the remission period, serving as a foundation for future research during the next escalation period. This baseline data provides valuable insights into the impact of relative calm on HCPs' attendance and can inform strategies for managing workforce continuity during times of heightened security threats. It is important to acknowledge that recall bias could be a potential limitation in this study, as participants answered the questionnaire 7 months after the event, which might have affected their accurate recollection of whether they worked during wartime. Moreover, to mitigate bias due to the COVID-19 pandemic on resilience and sense of danger, the study was conducted between peak waves of morbidity in Israel. Additionally, a recommendation for future research would be to conduct separate analyses to thoroughly account for the potential confounding effects of age and management position on the observed associations between resilience, sense of danger, and the outcomes during wartime among healthcare professionals.

Finally, it is important to acknowledge that this study relied solely on self-report data, which may be subject to desirability bias, even though the questionnaire was anonymous. Participants might still have been inclined to present themselves in a more socially desirable manner, potentially affecting the accuracy of the responses. To address this limitation and enhance the robustness of future research, it is recommended that studies explore the use of actual records of absenteeism and compare these objective figures to self-report data. This comparative approach can provide a more comprehensive understanding of attendance behaviors during wartime and mitigate potential biases associated with self-reporting.

## Data Availability

The datasets used and analysed during the current study are available from the corresponding author on reasonable request.
